# Urinary-derived extracellular vesicles reveal a distinct microRNA signature associated with the development and progression of Fabry nephropathy

**DOI:** 10.3389/fmed.2023.1143905

**Published:** 2023-03-23

**Authors:** Tina Levstek, Bojan Vujkovac, Andreja Cokan Vujkovac, Katarina Trebušak Podkrajšek

**Affiliations:** ^1^Laboratory for Translational Medical Biochemistry, Institute of Biochemistry and Molecular Genetics, Faculty of Medicine, University of Ljubljana, Ljubljana, Slovenia; ^2^Clinical Institute for Special Laboratory Diagnostics, University Children’s Hospital, University Medical Centre Ljubljana, Ljubljana, Slovenia; ^3^Centre for Fabry Disease, General Hospital Slovenj Gradec, Slovenj Gradec, Slovenia

**Keywords:** urinary miRnome profiling, microRNA, Fabry disease, nephropathy, urinary extracellular vesicles, biomarker, longitudinal study

## Abstract

**Introduction:**

Early initiation is essential for successful treatment of Fabry disease, but sensitive and noninvasive biomarkers of Fabry nephropathy are lacking. Urinary extracellular vesicles (uEVs) represent a promising source of biomarkers of kidney involvement. Among them, microRNAs (miRNAs) are important post-transcriptional regulators of gene expression that contribute to the development and progression of various kidney diseases. We aimed to identify uEV-derived miRNAs involved in the development and/or progression of Fabry nephropathy.

**Methods:**

Patients with genetically confirmed Fabry disease and matched control subjects were included. EVs were isolated from the second morning urine by size exclusion chromatography, from which miRNAs were extracted. miRNA urine exosome PCR panels were used to characterize the miRNA signature in a discovery cohort. Individual qPCRs were performed on a validation cohort that included chronological samples. We identified the target genes of dysregulated miRNAs and searched for potential hub genes. Enrichment analyses were performed to identify their potential function.

**Results:**

The expression of miR-21-5p and miR-222-3p was significantly higher in patients with stable renal function and those with progressive nephropathy compared with the corresponding controls. In addition, the expression of miR-30a-5p, miR-10b-5p, and miR-204-5p was significantly lower in patients with progressive nephropathy, however, in the chronological samples, this was only confirmed for miR-204-5p. Some of the identified hub genes controlled by the dysregulated miRNAs have been associated with kidney impairment in other kidney diseases.

**Conclusion:**

The miRNA cargo in uEVs changes with the development and progression of Fabry nephropathy and, therefore, represents a potential biomarker that may provide a new option to prevent or attenuate the progression of nephropathy. Furthermore, dysregulated miRNAs were shown to be potentially associated with pathophysiological pathways in the kidney.

## 1. Introduction

The kidneys are frequently affected in patients with Fabry disease (FD). In the Fabry Outcome Survey, baseline prevalence of kidney involvement was found in 59% of males and 38% of females in a cohort of 1,453 patients with FD (1). Fabry nephropathy is the result of complex pathogenic mechanisms initiated by the accumulation of globotriaosylceramide (Gb3) and its deacetylated form globotriaosylsphingosine (LysoGb3) (2), which occur in all types of renal cells (3). The clinical course of Fabry nephropathy is highly variable even between family members harboring the same pathogenic variant in the *GLA* gene (4). In addition, Fabry patients who develop kidney failure are more likely to have concomitant involvement of other major organ systems (5). Kidney involvement is characterized by albuminuria, proteinuria, and decreased glomerular filtration rate (GFR), but these markers lack sensitivity and are not sufficient to predict the progression of nephropathy at an early phase of the disease (6). Therefore, new sensitive and noninvasive biomarkers are urgently needed for the early detection of kidney damage and prediction of disease progression, which could improve the management of Fabry patients (6). Urine is a promising biofluid for the study of new biomarkers because urine samples can be obtained noninvasively, repeatedly, and in large quantities. However, most components of whole urine are derived from blood, making urine less suitable for detecting early structural changes in the kidneys (7).

In contrast, urinary extracellular vesicles (uEVs) originate from the nephrons and collecting ducts (8). EVs are small lipid membrane particles released into the extracellular space by various cell types (9). By transferring their molecular cargo between cells, they mediate various processes that depend on donor and recipient cells, their (patho)physiological state, and the microenvironment (10, 11). Therefore, the cargo of uEVs could provide valuable insights into kidney pathophysiology. In addition to other biomolecules, such as proteins, lipids, metabolites, etc., EV cargos also contain microRNAs (miRNAs) (12), these are short, single-stranded RNA molecules usually consisting of 18–26 nucleotides (13). In most cases, miRNAs exert their effects by binding to target mRNAs and either causing their degradation or inhibiting their translation (13).

Several studies have demonstrated an association between the expression of certain miRNAs and nephropathy (14–17). However, to our knowledge, miRNAs derived from uEVs have not been studied in Fabry patients. Therefore, we first performed a proof-of-concept study in which we demonstrated altered expression of some miRNAs (18). In the present study, we aimed to identify a panel of uEV miRNAs associated with the development and/or progression of Fabry nephropathy and the functions or pathways in which they converge. We also examined the longitudinal expression of seven candidate miRNAs over a 10-year period of disease progression. New insights into kidney injury at the molecular level may help us identify novel candidate biomarkers and elucidate pathophysiological processes in Fabry kidneys.

## 2. Materials and methods

### 2.1. Study participants

The discovery cohort consisted of 10 male Fabry patients and 10 age-matched male control subjects, whereas the validation cohort consisted of 33 Fabry patients and 33 age- and sex-matched control subjects. The genetic variants harbored by the patients in the *GLA* gene and clinical characteristics are listed in Supplementary Table S1. All patients were recruited from the Slovenian National Centre for Fabry disease in the General Hospital Slovenj Gradec. Urine and blood samples were collected at their annual follow-up examinations. Urine samples were transported on ice and centrifuged at 2,000 × *g* for 15 min within three hours to remove cells and debris; the supernatant was then transferred to new tubes and stored at −80 °C. Periodic urine samples from patients had already been collected and stored at −80 °C during their previous routine examinations; These were collected at intervals of about 2–3 years over the past decade. Clinical and laboratory examinations were performed as routinely scheduled. Kidney function was estimated using the Chronic Kidney Disease Epidemiology Collaboration (CKD-EPI) equation (19). Patients were divided into two cohorts: (1) Fabry patients with stable renal function and an estimated glomerular filtration rate (eGFR) slope < 3 mL/min/1.73 m^2^/year and (2) Fabry patients with progressive nephropathy and an eGFR slope > 3 mL/min/1.73 m^2^/year (20).

Inclusion criteria were age over 18 years, genetically confirmed FD, and at least three follow-up visits during the last five years. Exclusion criteria were uncontrolled hypertension, additional glomerular disease, dialysis, physical or psychological disease that might interfere with the normal conduct of the study, pregnancy at the time of enrollment or within the past 12 months, and current or recent history of alcohol or drug abuse.

One-time blood and urine samples were collected from the control subjects. Healthy control subjects were defined by the absence of a history of kidney disease or systemic disease that could cause nephropathy (i.e., diabetes, uncontrolled arterial hypertension), normal eGFR (age-adjusted), no albuminuria or proteinuria (urinary albumin-to-creatinine ratio (UACR) ≤ 3 g/mol, urinary protein-to-creatinine (UPCR) ≤ 20 g/mol), and no proliferative urine sediment.

The study protocol was approved by the Slovenian Ethics Committee for Research in Medicine (0120-260/2020/6, 0120-521/2020/3, and 0120-260/2020/12). All participants gave written informed consent in accordance with the Declaration of Helsinki.

### 2.2. Isolation of urinary extracellular vesicles and total RNA

uEVs were isolated from 8 mL of second-morning urine using a previously optimized method based on size exclusion chromatography (18, 21). Total RNA was extracted from uEVs following the protocol of the miRNeasy Mini Kit (Qiagen, Hilden, Germany) with slight modifications, as previously described (18). RNA quality and concentration were determined using NanoDrop One (Thermo Fisher Scientific, Waltham, MA, United States). RiboLock RNase Inhibitor (Thermo Fisher Scientific, Waltham, MA, United States) was added to each sample to prevent RNA degradation. Samples were stored at −80 °C in DNA low-binding tubes (Eppendorf, Hamburg, Germany) until further processing.

### 2.3. Reverse transcription of RNA to complementary DNA

Reverse transcription (RT) of total RNA to complementary DNA (cDNA) was performed using the miRCURY Locked Nucleic Acid (LNA) RT Kit (Qiagen, Hilden, Germany) according to the manufacturer’s instructions. For discovery analysis we prepared 50 μL reactions containing 10 μL of 5x miRCURY RT Reaction Buffer, 20 μL of RNase-free water, 5 μL of 10x miRCURY RT Enzyme Mix, and 15 μL of template RNA. For validation analysis, the volume of each reagent was five times smaller as the final volume of the reaction mixture was 10 μL. The reaction was performed in a GeneAmp^®^ PCR System 9,700 thermal cycler (Applied Biosystems, Waltham, MA, United States) and comprised an incubation step (60 min at 42 °C) and heat inactivation of the enzyme (5 min at 95 °C). cDNA was stored in a LoBind DNA plate (Eppendorf, Hamburg, Germany) at −80 °C and analyzed within four days.

### 2.4. miRNA profiling using urine exosome focus PCR panels in the discovery cohort

For miRNA profiling of the discovery cohort, the miRCURY LNA miRNA Urine Exosome Focus PCR Panel (Qiagen, Hilden, Germany) was used according to the manufacturer’s instructions. The panel included 87 miRNA LNA PCR primer sets targeting human miRNAs expressed in human urinary exosomes pre-aliquoted in 384-well plates. The reaction mix contained 2 mL of 2x miRCURY SYBR^®^ Green PCR Master Mix with added low ROX™ Reference Dye, 40 μL of cDNA, and 1960 μL of RNase-free water to give a final reaction volume of 10 μL per well. Each miRNA was measured in quadruplicate. An inter-plate calibrator spike-in control (UniSp3) was run six times per plate and was used to normalize the expression levels of all miRNAs included in each of the qPCR panels. A no template control was also included in each experiment. Amplification was performed using the QuantStudio 7 Flex Real-Time PCR System (Applied Biosystems, Waltham, MA, United States) under the following conditions: 95 °C for 2 min for heat activation, followed by 40 cycles of 95 °C for 10 s and 56 °C for 1 min. The signal was collected at the endpoint of each cycle. Following amplification, melting curve analysis was performed to verify specificity. The ramping rate was set to 0.05 °C/s for 60–95 °C. Raw quantification cycle (Cq) values were obtained from QuantStudio™ Real-Time PCR Software v1.3 (Applied Biosystems, Waltham, MA, United States) for further analysis.

### 2.5. Validation of miRNA expressions by quantitative PCR

Seven statistically significant differentially expressed miRNAs (DE-miRNAs) from the panel analysis with a fold change of at least 1.5 between patients and controls and three selected reference miRNAs were independently validated using miRCURY LNA miRNA PCR Assays (Qiagen, Hilden, Germany) according to the manufacturer’s instructions. Briefly, cDNA was diluted 1:30 with RNase-free water. The reaction mixture contained 5 μL of 2x miRCURY SYBR^®^ Green PCR Master Mix with added low ROX™ Reference Dye, 1 μL of resuspended PCR primer mix, 1 μL of RNase-free water, and 3 μL of cDNA template. qPCR conditions were as described for the panel analysis in section “miRNA profiling using urine exosome focus PCR panels in the discovery cohort.” Samples, inter-plate calibrator, and the no template control were measured in triplicate. In addition to samples from Fabry patients and their corresponding control subjects, three to five chronological samples per patient were also included in the analysis. A total of 152 samples from 33 Fabry patients and 33 from control subjects were included. For the validation cohort, we set stricter criteria; only miRNAs with cut-off values with at least a two-fold change and false discovery rate (FDR) < 0.05 were considered DE-miRNAs.

### 2.6. Pre-processing of quantitative PCR data

To correct for any inter-run variations in the raw data of the discovery cohort, inter-plate calibration was performed using the following equation: Cq_norm_ = Cq(miRNA) − average Cq(UniSp3). NormFinder (22) was used to find the most stably expressed miRNAs between groups and these were subsequently used as reference miRNAs. The miRNA Cq values were normalized *via* the following formula: ΔCq = average Cq (miRNA) − average Cq (reference miRNAs). The miRNA-specific ΔCq values for all samples in the same group were averaged. The ΔΔCq value was calculated by subtracting the miRNA-specific average ΔCq value of each group from the corresponding value of the control group. Fold change was calculated according to the equation 2^−ΔΔCq^ (23).

### 2.7. Target gene prediction and hub gene identification

Target gene prediction and hub gene identification were performed separately for upregulated and downregulated miRNAs. Target genes of DE-miRNAs were predicted using two databases of experimentally validated targets [miRTarBase v9.0 (24), Diana-TarBase v8 (25)] and two of predicted targets [miRDB v6.0 (26), TargetScan Human v8.0 (27)], as these databases are widely used and frequently updated. Predicted targets were excluded if they had a target score below 80 in miRDB or a cumulative weighted context++ score above −0.2 in TargetScan. Target genes predicted by at least two databases were then further considered.

The protein–protein interaction (PPI) network of target genes was generated using the Search Tool of the Retrieval of Interacting Genes database (STRING v11.5) (28). The minimum required interaction score was set to 0.40. The PPI network was visualized using Cytoscape v3.9.1 software (29), followed by a search for hub genes with 11 topological analysis methods using the cytoHubba v0.1 plug-in (30). The top 20 genes were selected for each method, and those found in the intersection of at least six methods were selected as hub genes.

### 2.8. Functional enrichment analysis of hub genes

Hub gene candidates were subjected to gene ontology (GO) enrichment analysis and included three categories, namely biological process (BP), cellular component (CC), and molecular function (MF). The Reactome database was used to investigate the functions of a biological system. The Gene annotation co-occurrence discovery classification system (GeneCodis4) was used for analysis (31). The Wallenius test was applied to avoid bias in miRNA functional enrichment analysis (32). An FDR < 0.05 was considered statistically significant.

### 2.9. Statistical analysis

Descriptive statistics were used to summarize the characteristics of the study population. The Shapiro–Wilk test was used to test normality. Data were expressed as medians (interquartile range) for continuous variables and frequencies (percentage) for categorical variables. Continuous variables were compared between groups using the Mann–Whitney *U* test, whereas the Chi-square test was used for categorical variables. Spearman’s Rho coefficient was used to assess the correlation between continuous variables. The diagnostic performance of miRNAs was evaluated by calculating their sensitivity and specificity using receiver operating characteristic curves (ROC). Statistical analyses were performed using SPSS version 27.0 (IBM Corporation, Armonk, NY, United States). A *p*-value <0.05 was considered significant. When indicated, correction for multiple testing was performed using FDRs.

Linear mixed effects models were built using the lme4 package in R (version 4.2.1, R core team) (33, 34). Time and group (stable renal function vs. progressive nephropathy) were included as fixed effects in the model, whereas a random intercept for subject was included to account for within-subject correlations. To approximate the outcome to a Gaussian distribution, miRNA expression was converted to a logarithmic scale. The Satterthwaite approximation of the lmerTest package was used to calculate degrees of freedom (35). The residual plots were used to test for deviations from homoscedasticity and normality.

## 3. Results

### 3.1. Discovery cohort characteristics

To identify miRNA candidates involved in the development and progression of Fabry nephropathy, ten male Fabry patients and ten age- and sex-matched control subjects were included in the discovery cohort. Five patients with stable renal function had a median eGFR slope of −0.62 (−0.75 to −0.26) mL/min/1.73 m^2^/year and daily proteinuria of 0.22 (0.12–0.36) g/day; they differed significantly in eGFR and serum creatinine, which could be an indicator of hyperfiltration in some of the Fabry patients. In five patients with progressive nephropathy, the median eGFR slope was −4.8 (−7.0 to −4.2) mL/min/1.73 m^2^/year and daily proteinuria was 1.27 (0.59–2.15) g/day. All patients were on disease-specific therapy (DST) (enzyme replacement therapy or chaperones) with a median of 10.1 (6.2–11.5) years in patients with stable renal function and 6.0 (3.0–10.3) years in those with progressive nephropathy. Detailed characteristics of the discovery cohort are presented in Table 1.

**Table 1:** Discovery cohort characteristics.

Variable	FD-SRF (*n* = 5)	Control group (*n* = 5)	*p*-value	FD-PN (*n* = 5)	Control group (*n* = 5)	*p*-value
Age (years)	42.1 (27.0–43.9)	41.7 (26.4–45.2)	1.000	53.7 (49.9–56.9)	51.3 (49.9–56.6)	0.841
eGFR (mL/min/1.73 m^2^)	119 (112–126)	104 (92–107)	**0.016**	30 (17–71)	95 (86–101)	**0.008**
UPCR (g/mol)	39.8 (16.1–91.1)	4.5 (3.7–5.1)	**0.016**	115.4 (42.6–257.3)	7.3 (6.1–10.3)	**0.016**
UACR (g/mol)	20.1 (1.2–56.6)	0.6 (0.4–0.7)	0.111	105.5 (51.2–244.3)	0.4 (0.4–1.0)	**0.008**
Serum Cr (μmol/L)	59 (55–76)	86 (74–95)	**0.032**	208 (106–380)	80 (75–90)	**0.008**

FD-SFR, Fabry patients with stable renal function; FD-PN, Fabry patients with progressive nephropathy; eGFR, estimated glomerular filtration rate; UPCR, urinary protein-to-creatinine ratio; UACR, urinary albumin-to-creatinine ratio; Cr, creatinine. Bold indicates statistically significant results.

### 3.2. Identification of differentially expressed miRNAs in the discovery cohort

Of the 87 miRNAs included in the panel, 81 miRNAs gave consistent measurements with Cq < 35 in more than 80% of the samples. Six miRNAs (miR-34a-5p, miR-126-3p, miR-133a-3p, 145-5p, miR-187-3p, and miR-301a-3p) were excluded from further analysis because they could not be detected in several samples. Using NormFinder, miR-17-5p, miR-20a-5p, and miR-106a were found to be the most stably expressed miRNAs among the different groups and were thus selected as reference miRNAs for normalization.

**Figure 1:**
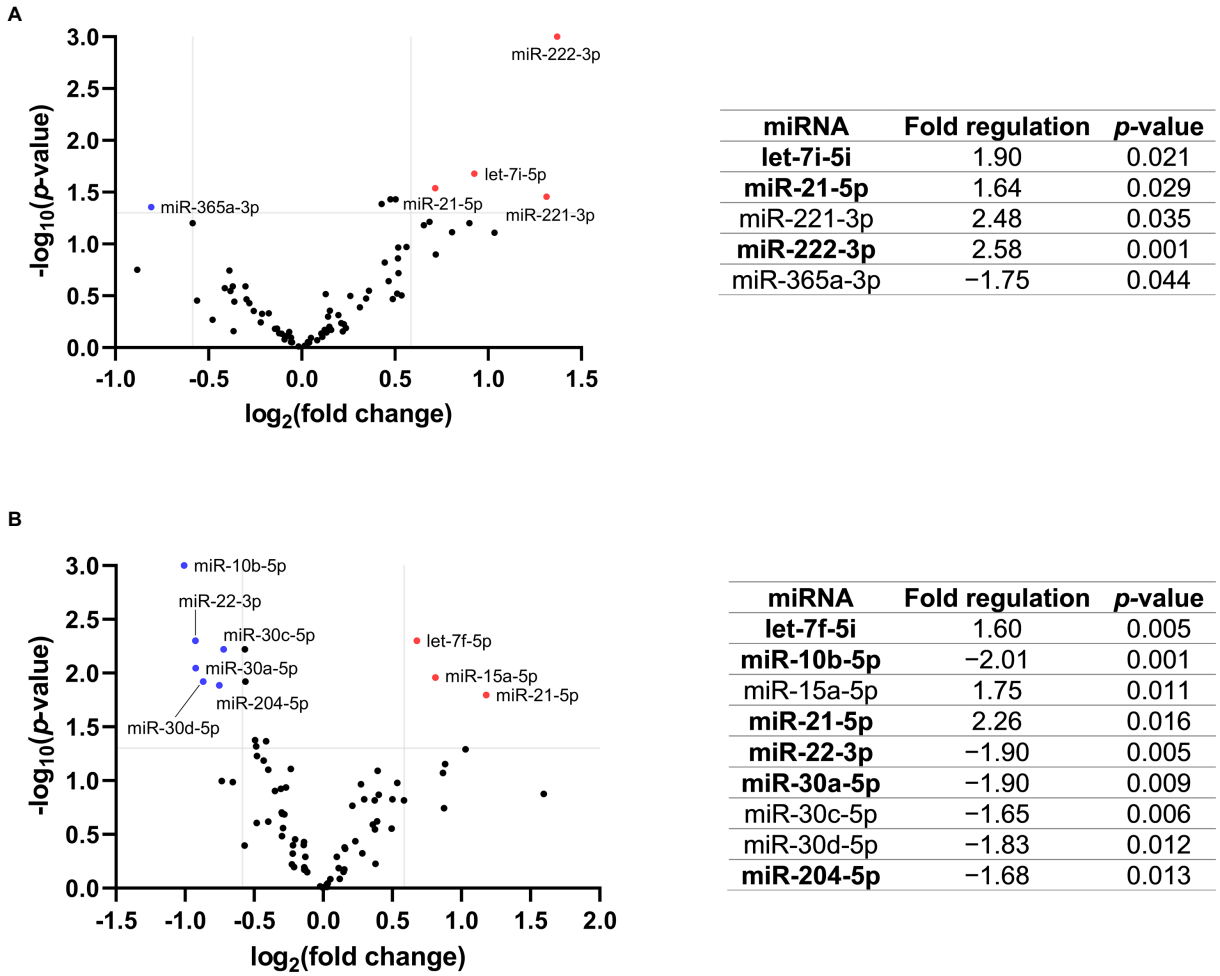
Volcano plot with the corresponding table showing the comparison of miRNA expression of Fabry patients with **(A)** stable renal function (*n* = 5) or **(B)** progressive nephropathy (*n* = 5) with the corresponding control group (*n* = 5). The blue dot indicates downregulated and the red dots indicate upregulated miRNAs. The grey lines represent the cut-off criteria for significant regulation. Only miRNAs with a fold change >1.5 and a *p-*value <0.05 are listed in the table. The miRNAs marked in bold were selected for validation.

In the discovery cohort, Fabry patients with stable renal function had significantly higher expression of let-7i-5p, miR-21-5p, miR-221-3p, and miR-222-3p and significantly lower expression of miR-365a-3p compared with control subjects (Figure 1A).Fabry patients with progressive nephropathy had significantly higher expression of let-7f-5p, miR-15a-5p, and miR-21-5p, and decreased expression of miR-10b-5p, miR-22-3p, miR-30a-5p, miR-30c-5p, miR-30d-5p, and miR-204-5p compared with control subjects (Figure 1B).

Of the 13 potential DE-miRNAs identified in the discovery cohort, seven were selected for further validation based on their fold change and *p*-value. Of note, after FDR correction, the difference in miRNA expression was no longer statistically significant. Only one miRNA from each miRNA family was selected for validation because miRNAs from the same family often serve a similar biological function due to their similar sequence.

### 3.3. Validation cohort characteristics

The validation cohort included 33 Fabry patients and 33 age- and sex-matched control subjects. Of the 25 patients with stable renal function, 20 were women and 5 were men. On the other hand, four men and four women had progressive nephropathy. Nine patients (five men and four women) with stable renal function received DST for a median of 14.5 (10.8–16.1) years. Their eGFR slope was −1.05 (−1.64 to −0.55) mL/min/1.73 m^2^/year and daily proteinuria was 0.11 (0.09–0.28) g/day. All patients except one with progressive nephropathy were on DST for a median of 11.0 (5.6–14.5) years. They had an eGFR slope of −4.28 (−6.26 to −3.49) mL/min/1.73 m^2^/year and daily proteinuria of 0.21 (0.16–1.32) g/day. Detailed characteristics of the validation cohort are presented in Table 2.

**Table 2:** Validation cohort characteristics.

Variable	FD-SRF (*n* = 25)	Control group (*n* = 25)	*p*-value	FD-PN (*n* = 8)	Control group (*n* = 8)	*p*-value
Male (%)	5 (20)	5 (20)	1.000	4 (50)	4 (50)	1.000
Age (years)	46.8 (35.4–55.8)	46.6 (34.7–56.2)	0.961	56.3 (47.6–60.1)	57.9 (48.5–60.4)	0.798
eGFR (mL/min/1.73 m^2^)	103 (96–116)	96 (89–107)	0.091	57 (24–88)	99 (90–100)	**0.043**
UPCR (g/mol)	13.5 (9.7–47.9)	5.6 (4.1–7.1)	**<0.001**	52.8 (8.7–212.8)	7.1 (3.8–7.3)	**0.007**
UACR (g/mol)	2.0 (0.8–32.3)	0.7 (0.4–0.9)	**<0.001**	147.0 (1.0–258.4)	0.6 (0.4–0.8)	**0.010**
Serum Cr (μmol/L)	63 (57–68)	69 (65–78)	**0.001**	110 (79–245)	73 (66–84)	**0.043**

FD-SFR, Fabry patients with stable renal function; FD-PN, Fabry patients with progressive nephropathy; eGFR, estimated glomerular filtration rate; UPCR, urinary protein-to-creatinine ratio; UACR, urinary albumin-to-creatinine ratio; Cr, creatinine. Bold indicates statistically significant results.

### 3.4. Validation of selected miRNAs using quantitative PCR

**Figure 2:**
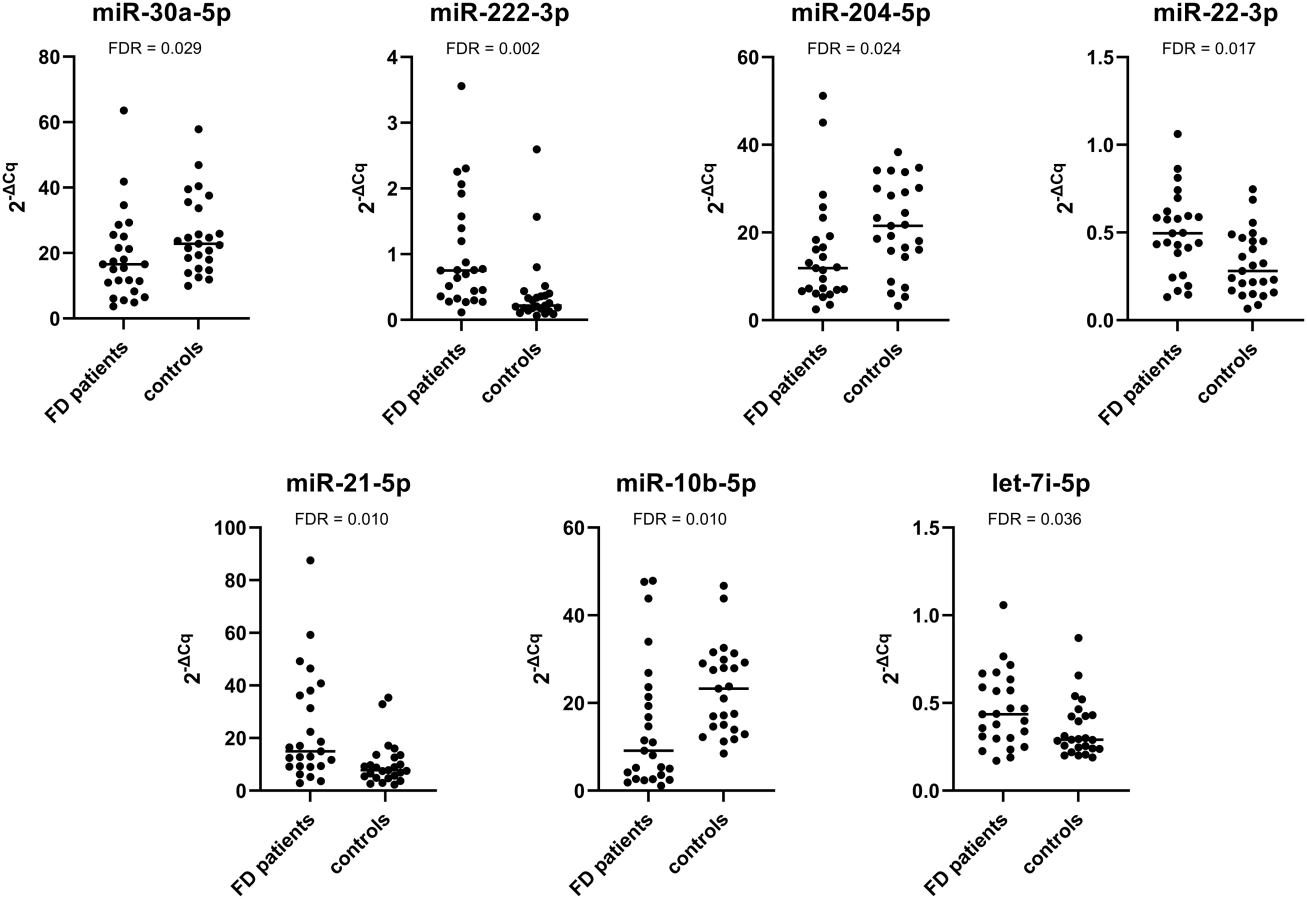
Relative expression of selected miRNAs in the validation cohort composed of Fabry patients with stable renal function and their matched controls.

The expression of miR-222-3p and miR-21-5p was significantly upregulated in Fabry patients with stable renal function compared with the corresponding control subjects. The expression of the other miRNAs was not significantly different between patients and control subjects. The relative expression profiles of all miRNAs are shown in Figure 2.

**Figure 3:**
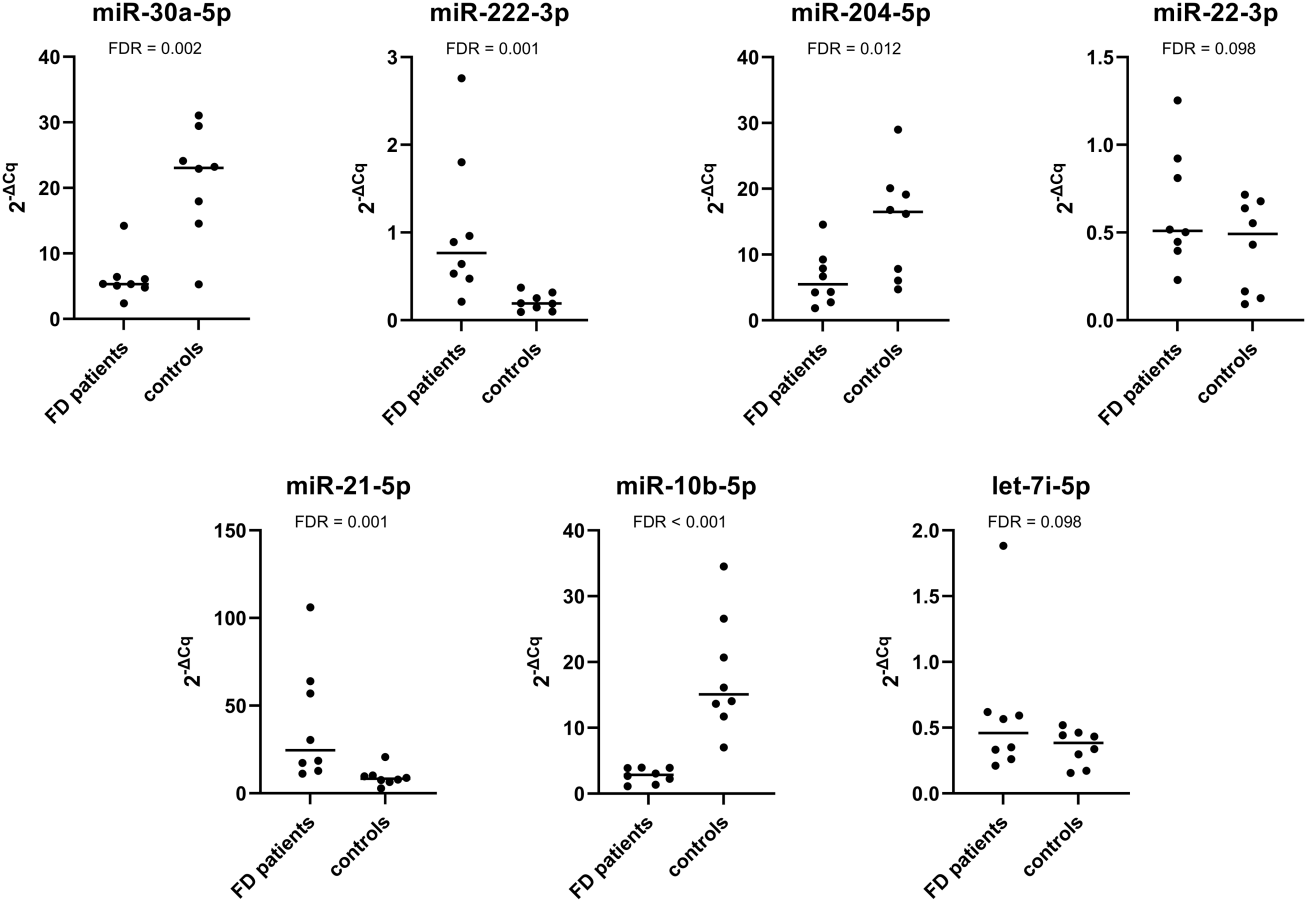
Relative expression of selected miRNAs in the validation cohort composed of Fabry patients with progressive nephropathy and their matched controls.

The expression of miR-222-3p and miR-21-5p was significantly upregulated in patients with progressive nephropathy compared with control subjects. Additionally, the expression of miR-30a-5p, miR-204-5p, and miR-10b-5p was significantly downregulated. The relative expression profiles of all miRNAs are shown in Figure 3.

**Figure 4:**
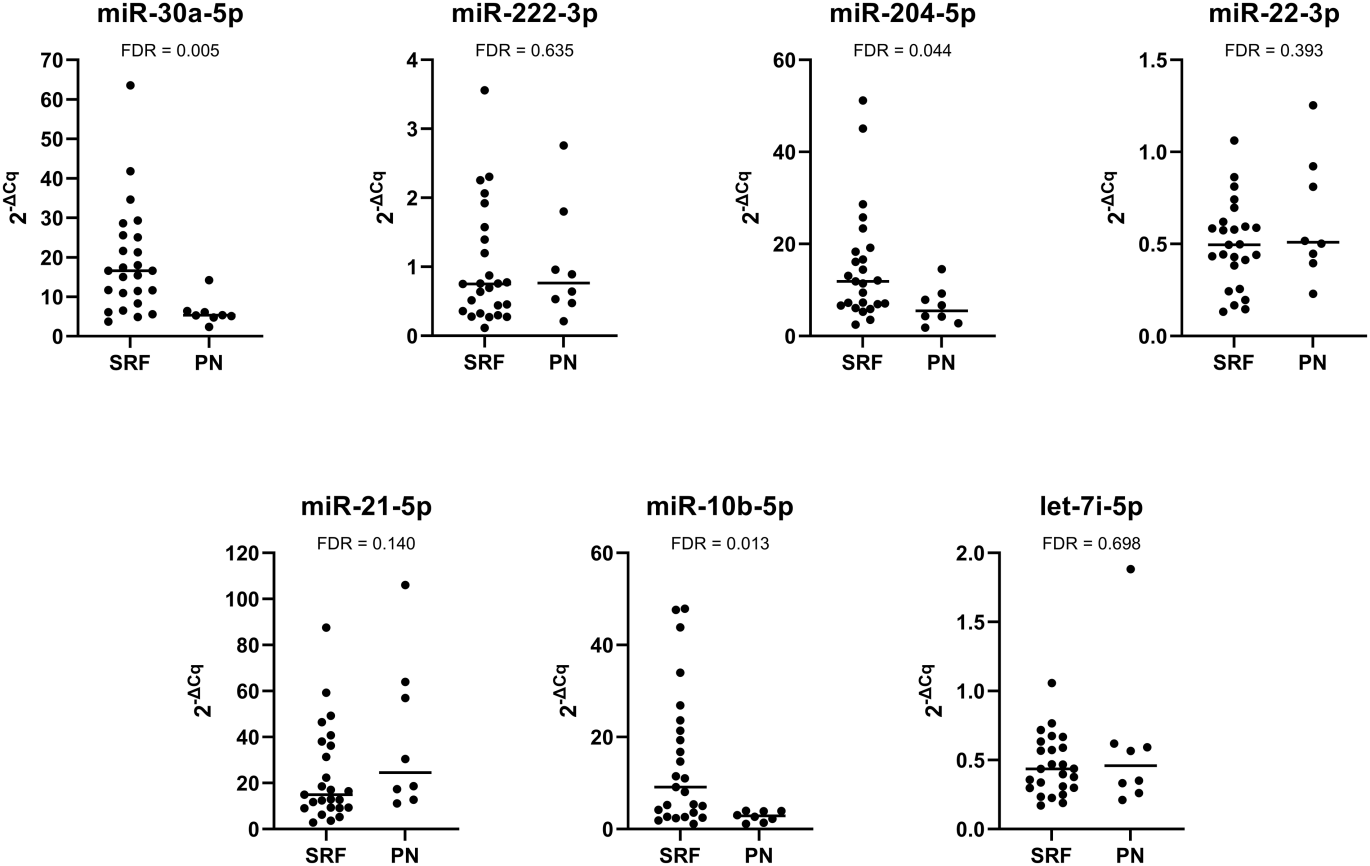
Comparison of relative expression of selected miRNAs between Fabry patients with stable renal function (SRF) and progressive nephropathy (PN).

We also investigated the difference in miRNA expression between patients with stable renal function and progressive nephropathy. The expression of miR-30a-5p, miR-204-5p, and miR-10b-5p was significantly downregulated in patients with progressive nephropathy than in patients with stable renal function. The expression of miR-21-5p was higher in patients with progressive nephropathy, but the difference was not statistically significant. The expression of miR-222-3p was also not significantly different. The relative expression profiles of all miRNAs are shown in Figure 4.

### 3.5. Evaluation of miRNA expression in the chronological samples with linear mixed models

The summary of the linear mixed models for each of the seven miRNA analyses in chronological samples is shown in Supplementary Table S2. Interestingly, a statistically significant effect was only seen for miR-204-5p expression (*p* = 0.005), where the logarithm of miRNA expression decreased on average by 0.012 ± 0.004 per year. For other miRNAs, no statistically significant differences in miRNA expression over time were detected.

We also found that group status significantly affected the expression of miR-30a-5p, miR-204-5p, and miR-10b-5p (*p* < 0.001, *p* = 0.002, and *p* = 0.019, respectively). The expression of all three miRNAs was lower in patients with progressive nephropathy compared with those with stable renal function. On average, patients with progressive nephropathy had 0.349 ± 0.094 units lower expression of mi-30a-5p, 0.273 ± 0.110 units for miR-204-4p, and 0.485 ± 0.147 units for miR-10b-5p. The influence of group was also evident in miR-21-5p expression, where patients with progressive nephropathy had a higher expression, but this difference was not statistically significant (*p* = 0.095).

### 3.6. Evaluation of differentially expressed miRNAs as potential biomarkers of nephropathy

**Figure 5:**
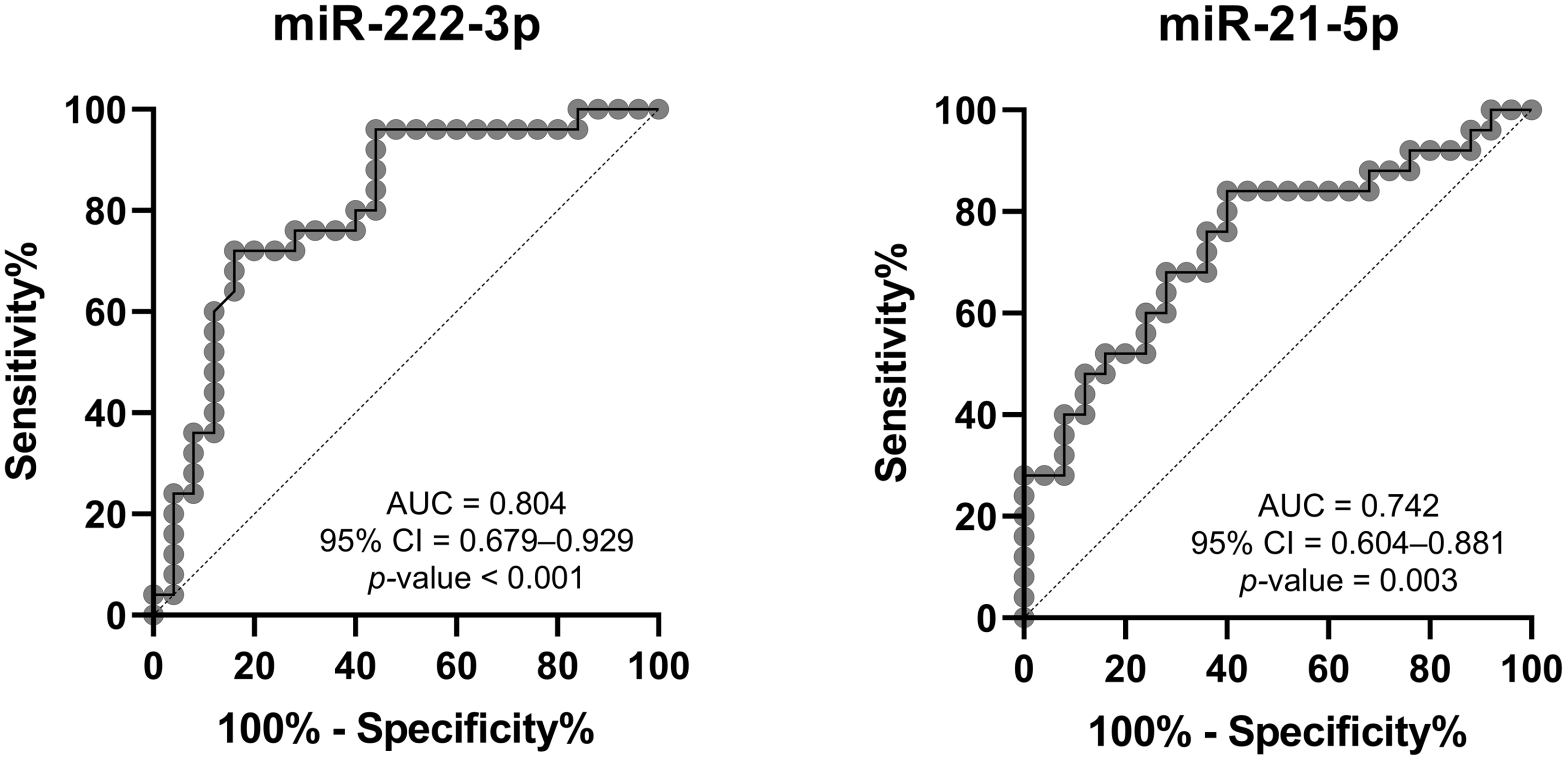
Receiver operating characteristic analysis of DE-miRNAs to discriminate between Fabry patients with stable renal function and matched controls. AUC, area under the curve; CI, confidence interval.

Receiver operating curves (ROC) and areas under the curve (AUC) were used to evaluate the feasibility of using uEV-derived miRNAs as diagnostic tools for Fabry nephropathy. As shown in Figure 5, miR-222-3p and miR-21-5p presented moderate discriminatory power, with an AUC of 0.804 and 0.742, respectively, between patients with stable renal function and control subjects. When both miRNAs were considered together, the analysis yielded an AUC of 0.789.

**Figure 6:**
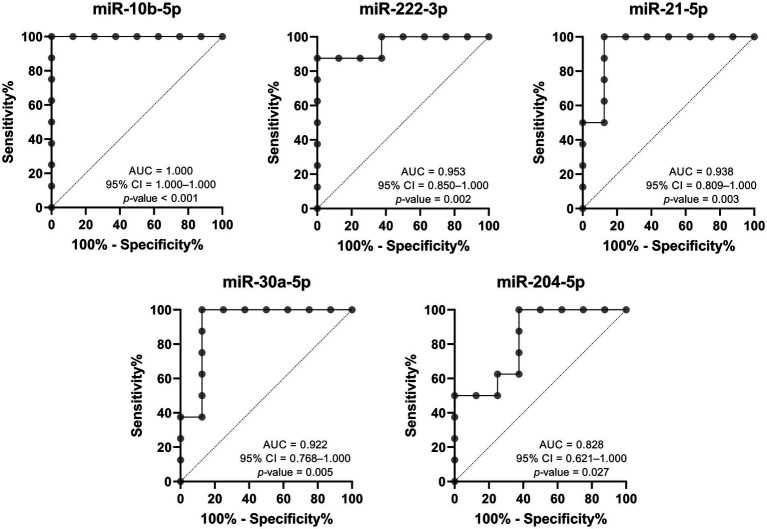
Receiver operating characteristic analysis of DE-miRNAs to discriminate between Fabry patients with progressive nephropathy and matched controls. AUC, area under the curve; CI, confidence interval.

On the other hand, the discriminatory power between patients with progressive nephropathy and their control subjects was high and statistically significant for all five DE-miRNAs, with the AUC of miR-10b-5p being the highest, as shown in Figure 6. The evaluation was also performed for the combination of all upregulated miRNAs, all downregulated miRNAs, and all five DE-miRNAs. The combined analysis resulted in an AUC of 1.000 for all three combinations. Although the discriminatory power is high, we would like to emphasize that the sample size was small and therefore the results should be interpreted with caution.

### 3.7. Correlation between the expression of differentially expressed miRNAs and biochemical parameters

As shown in Table 3, Spearman’s Rho correlation analysis revealed a positive correlation between UPCR and the expression of miR-222-3p, miR-22-3p, and miR-21-5p. On the other hand, there was a negative correlation between UPCR and the expression of miR-204-5p and miR-10b-5p. The analysis also showed a positive correlation between UACR and the expression of miR-222-3p, miR-21-5p, and let-7i-5p; were negatively associated.

**Table 3:** Correlation between biochemical parameters and expression of miRNAs derived from uEVs in a validation cohort.

miRNA	UPCR		UACR		Serum Cr		eGFR	
	Rho	*p-*value	Rho	*p-*value	Rho	*p-*value	Rho	*p-*value
miR-30a-5p	−0.092	0.489	−0.067	0.632	0.027	0.833	−0.054	0.678
miR-222-3p	0.465	**<0.001**	0.404	**0.002**	−0.284	**0.025**	−0.116	0.371
miR-204-5p	−0.284	**0.029**	−0.211	0.126	0.100	0.441	−0.066	0.609
miR-22-3p	0.359	**0.005**	0.195	0.158	−0.070	0.588	−0.224	0.080
miR-21-5p	0.572	**<0.001**	0.405	**0.002**	0.063	0.628	−0.305	**0.016**
miR-10b-5p	−0.296	**0.023**	−0.195	0.158	0.060	0.643	−0.054	0.678
let-7i-5p	0.232	0.076	0.290	**0.033**	−0.172	0.180	−0.153	0.234

UPCR, urinary protein-to-creatinine ratio; UACR, urinary albumin-to-creatinine ratio; Cr, creatinine; eGFR, estimated glomerular filtration rate. Bold indicates statistically significant results.

### 3.8. Differentially expressed miRNA target gene prediction, protein–protein interaction network construction, and identification of hub genes

To investigate the role of DE-miRNAs in the development and progression of Fabry nephropathy, we analyzed their potential target genes. We predicted 922 target genes for upregulated DE-miRNAs and 1719 for downregulated DE-miRNAs. PPI networks were constructed to determine the interaction relationships between the proteins expressed by these potential target genes. Genes targeted by upregulated miRNAs formed a network with 5,619 edges, whereas genes targeted by downregulated miRNAs formed a network with 15,135 edges.

Hub genes were identified by 11 topological analysis methods, with the top 20 genes being selected from each method (Supplementary Tables S3, S4). Genes found in the intersection of at least six methods were selected as hub genes. For the upregulated miRNAs, the following hub genes were identified: *MYC*, *EGFR*, *ESR1*, *PTEN*, *HSPA8*, *BRCA1*, *STAT3*, *MDM2*, *HIF1A*, *SOX2*, *VEGFA*, *PIK3R1*, *DICER1*, and *FOS*. For the downregulated miRNAs, the following hub genes were identified: *TP53*, *CTNNB1*, *EGFR*, *HSP90AA1*, *PTEN*, *JUN*, *HSPA5*, *MAPK1*, *SIRT1*, *CDKN2A*, *PIK3CA*, *CREB1*, *CASP3*, *IL1B*, and *IGF1R*.

### 3.9. Go function and KEGG pathway enrichment analysis

**Figure 7:**
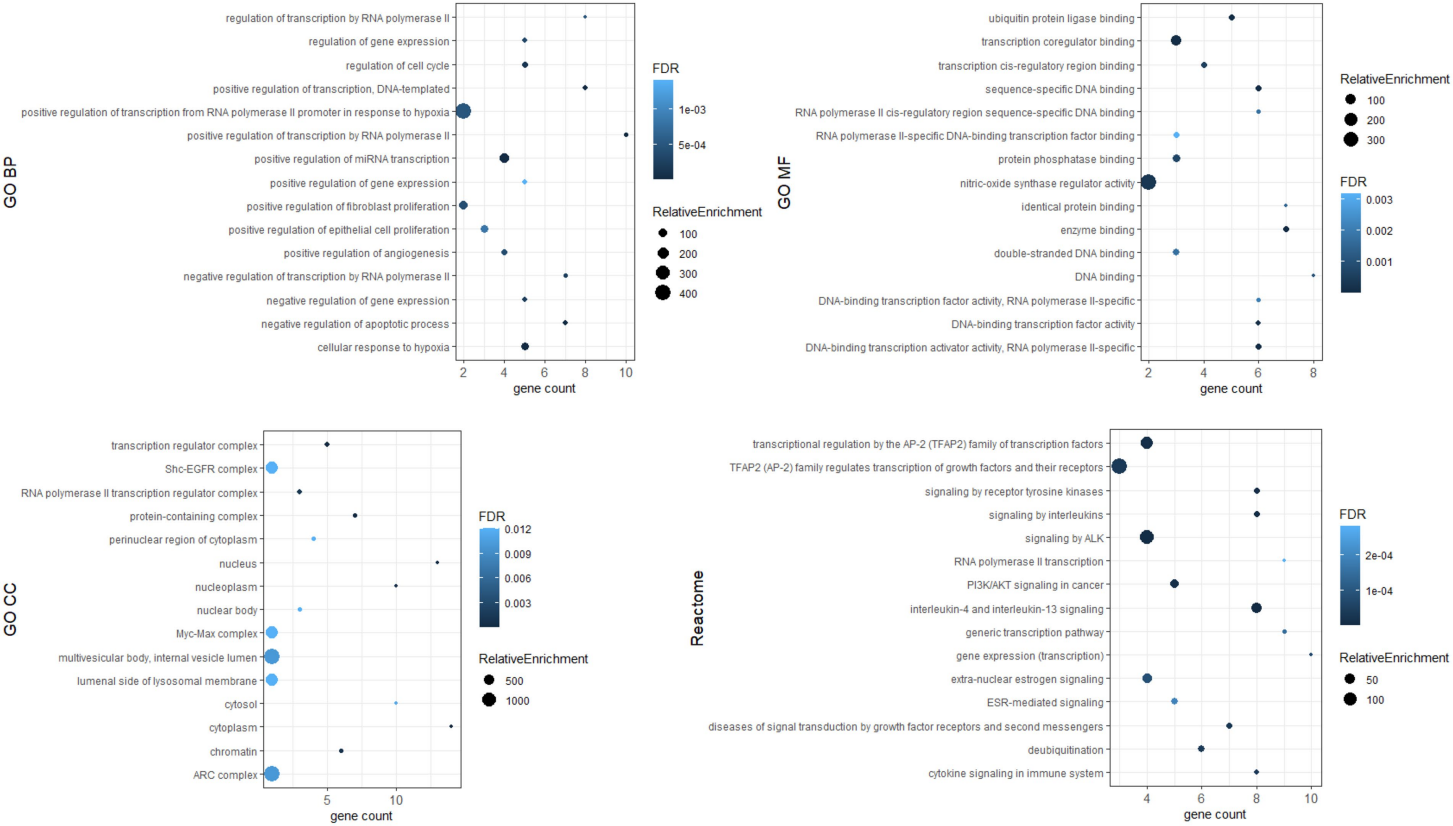
Top 15 terms for hub genes targeted by upregulated miRNAs.

**Figure 8:**
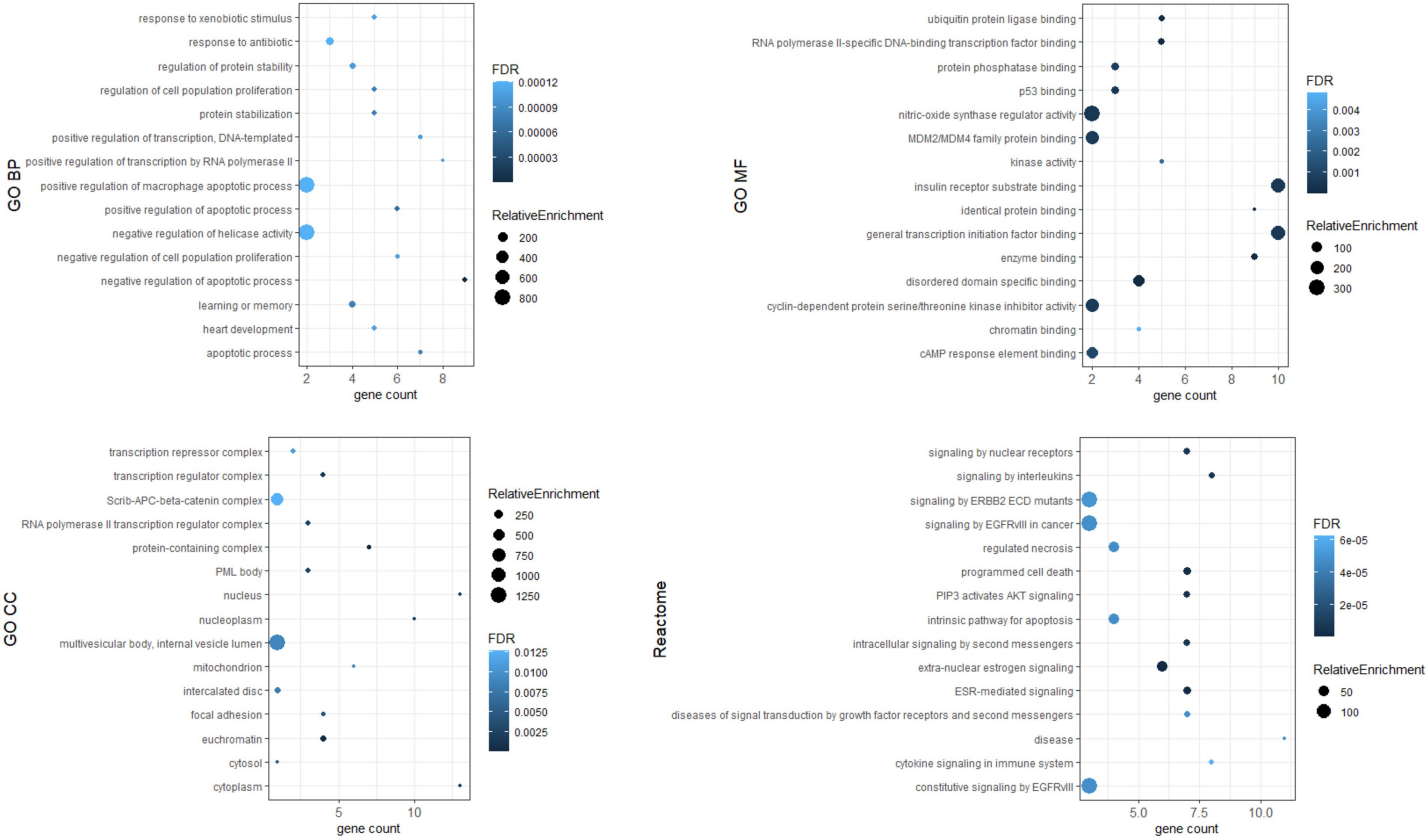
Top 15 terms for hub genes targeted by downregulated miRNAs.

To investigate the biological functions of the identified hub genes targeted by the DE-miRNAs, GO function and Reactome pathway enrichment analysis were performed. Figure 7 shows the top 15 terms for each category for genes targeted by upregulated miRNAs, and Figure 8 shows the top 15 terms for each category for genes targeted by downregulated miRNAs.

## 4. Discussion

Increasing evidence suggests that miRNAs play an important role as master regulators of gene expression not only in the development, homeostasis, and physiological functions of the kidney but also in the development and progression of nephropathy (36). miRNA profiling has been proposed as a biomarker discovery tool, but to our knowledge, it has not yet been performed in uEVs of Fabry patients. In the present study, we performed comprehensive profiling of miRNAs from the uEV cargo of Fabry patients and control subjects to characterize differentially expressed miRNAs that may be associated with the development and progression of Fabry nephropathy.

We used a two-step protocol that allowed the screening of 87 uEV-derived miRNAs and subsequent validation of selected dysregulated miRNAs. We identified five miRNAs with significant changes in expression in Fabry patients with stable renal function compared with control subjects, or in Fabry patients with progressive nephropathy compared with control subjects. In both, patients with progressive nephropathy and those with stable renal function, miR-21-5p and miR-222-3p expression was upregulated compared with the corresponding control subjects, suggesting that these miRNAs are probably important in the early stages of the disease process. Based on several studies, miR-21-5p is considered to be one of the most important factors in kidney fibrosis and has been reported to be upregulated in various kidney diseases, biofluids, and kidney tissues (16, 37–40). Overexpression of miR-21-5p has been shown to lead to transforming growth factor (TGF)-β-induced epithelial-mesenchymal transition by targeting Smad7 and the phosphatase and tensin homolog (PTEN). In turn, miR-21-5p is upregulated by TGF-β in tubular epithelial cells and promotes renal fibrosis (41, 42); fibrosis also being one of the key features of FD (43). Additionally, miR-21-5p silencing prevented podocyte loss and reduced albuminuria in mice (38). The role and mechanisms of miR-222-3p in nephropathy are less known. miR-222-3p expression had been shown to be upregulated in urinary exosomes of patients with chronic kidney disease (CKD) compared with healthy controls (44) and in the urine of type 1 diabetic patients with persistent compared with intermittent albuminuria (45). Both upregulated miRNAs were positively correlated with UPCR and UACR, whereas only miR-21-5p was negatively correlated with eGFR. miR-21-5p upregulation and negative correlation with eGFR were previously reported in glomerular and proximal tubules from patients with diabetic kidney disease (DKD), focal segmental glomerulosclerosis, and membranoproliferative glomerulonephritis (46), and also in uEVs from patients with DKD (16). This suggested that miR-222-3p and miR-21-5p may be involved not only in the development but also the progression of nephropathy. However, miRNA expression analysis in chronological samples did not reveal significant differences over time. A trend toward increased miR-21-5p expression was observed, but was not statistically significant. Although the miR-21-5p could be considered only as a marker of proteinuria and reduced glomerular filtration rate, its expression was higher in patients with progressive nephropathy than in patients with stable renal function, but the difference was not statistically significant. This may be because our group of patients with progressive nephropathy is small. Therefore, further studies with larger cohorts are needed to confirm our results.

In contrast, miR-30a-5p, miR-10b-5p, and miR-204-5p were significantly downregulated in patients with progressive nephropathy compared with control subjects. All three miRNAs exhibited an AUC greater than 0.80 for the diagnosis of progressive nephropathy. Moreover, the expression of miR-10b-5p and miR-204-5p was significantly negatively associated with UPCR. Therefore, they may be promising diagnostic indicators of nephropathy progression. As seen in other studies, the miR-30 family is abundantly expressed in human glomerular podocytes, where it plays a role in podocyte health and maintenance of cytoskeletal integrity (47, 48). TGF-β has been shown to downregulate miR-30 expression in podocytes. Overexpression of miR-30 ameliorates TGF-β-induced apoptosis of podocytes, while its knockdown exacerbates podocyte injury (47, 48). The miR-30 family exerts a protective role by inhibiting Notch1 and p53, which are involved in podocyte injury (48). Exposing human podocytes to LysoGb3, an accumulation product in FD, contributes to the activation of Notch1 signaling and leads to Notch1 upregulation (49). In addition, the miR-30 family regulates the calcium/calcineurin signaling pathway, the overexpression of which leads to cytoskeletal damage and podocyte apoptosis (50, 51). Downregulation of the miR-30 family was detected in the glomeruli and proximal tubules of DKD patients (46). In contrast to our results, increased expression of miR-30a was found in the uEVs of patients with type 2 diabetic kidney disease compared with healthy subjects (52), and in patients with overt albuminuria compared with normoalbuminuric patients with type 1 diabetes (53). The same study also reported increased miR-10b-5p expression in the urine of patients with persistent albuminuria compared with those with intermittent albuminuria (53). miR-204 is one of the most abundantly expressed miRNAs in the kidneys and plays a protective role in kidney injury (54). A significant decrease in miR-204-5p expression was found in the kidneys of patients with hypertensive nephrosclerosis and diabetic nephropathy. The authors suggested that miR-204-5p may protect the kidney from fibrotic damage by targeting SHP2 and suppressing the activation of STAT3. Studies in renal tubular cells found that miR-204-5p inhibits inflammation and chemokine synthesis by modulating the IL6/IL6 receptor axis (55). Moreover, miR-204 is involved in the regulation of epithelial-mesenchymal transition (56). Consistent with our results, the expression of miR-204-5p was shown to be downregulated in renal biopsies from patients with progressive CKD (57). Furthermore, our analysis of chronological samples showed a decreasing expression of miR-204-5p over time, suggesting that its protective role deteriorated during the progression of Fabry nephropathy. The DE-miRNAs identified are likely involved in molecular signaling pathways associated with Fabry nephropathy, but further studies are required to elucidate their function in the pathogenesis of Fabry nephropathy.

Dysregulated miRNA may affect the expression of target genes. We identified hub genes whose dysregulated expression likely plays a key role in the pathogenesis of Fabry nephropathy. Some proteins of these genes have already been associated with pathophysiological processes in the kidney: in tubulointerstitial fibrosis (c-MYC, PTEN, STAT3, HIF-1α, PT53) (58–62); podocyte injury (EGFR, PT53, CTNNB1, CREB1) (60, 63–65); epithelial-mesenchymal transition (PTEN) (66); and glomerulosclerosis (DICER1, IL1β) (67, 68). ESR1, PTEN and MDM2 have been shown to protect podocytes from injury (69–71) and SIRT1 has been attributed a protective role in the kidney (72). In addition, functional enrichment analysis was performed in which pathways such as apoptosis, inflammation, cytokine signaling, fibroblast proliferation, nitric oxidase synthase activity etc. were enriched. These signaling pathways had already been associated with the development and progression of nephropathy (73); they also significantly contribute toward FD pathogenesis (74–77). Therefore, our findings provided a knowledge base for ongoing studies to validate the functional role of dysregulated miRNAs and their target genes in the pathogenesis of Fabry nephropathy.

There are several limitations of the study that must be considered. First, a relatively small number of patients were included, which is a common drawback of rare disease studies. However, our study cohort was strictly defined, and only patients who had been meticulously followed for at least five years were included. In addition, only male patients were included in the discovery cohort to ensure as much homogeneity as possible. We also included sex- and age-matched control subjects, which is very important as the expression of some miRNAs changes with age. Second, the clinical importance of DST and adjuvant therapy on miRNA expression is uncertain and difficult to assess. Third, the standardization of functional enrichment analysis is still pending (78). Therefore, more frequently studied genes and diseases, such as cancer, are more likely to be significantly enriched. Thus, the functional enrichment results are theoretical and require further confirmatory studies. Studies examining miRNA changes longitudinally are extremely rare, although they could provide valuable insights into disease pathogenesis. Therefore, apart from the well-defined study group, one of the major strengths of our study was the assessment of miRNA expression in chronological urine samples.

## 5. Conclusion

We report the discovery of a five-miRNA profile showing aberrant expression in the uEVs of Fabry patients compared with control subjects. Some of these miRNAs were associated with signaling pathways known to be important in the development and progression of nephropathy in other diseases such as CKD and diabetic nephropathy. DE-miRNAs derived from uEVs can be used not only as molecular signatures of different clinical phenotypes in Fabry nephropathy, but also as early biomarkers of alterations in biological processes in Fabry kidneys. However, further studies are needed to elucidate the critical role of the miRNAs identified in the development and progression of Fabry nephropathy.

## Data availability statement

The raw data supporting the conclusions of this article will be made available by the authors, without undue reservation.

## Ethics statement

The studies involving human participants were reviewed and approved by the Slovenian Ethics Committee for Research in Medicine (0120-260/2020/6, 0120-521/2020/3, and 0120-260/2020/12). The patients/participants provided their written informed consent to participate in this study.

## Author contributions

KTP acquired the funding and supervised the project. TL and KTP conceived and planned the experiments. BV and ACV executed the clinical part of the study and curated clinical data. TL carried out the experiments, performed the statistical analysis, visualized the data, and wrote the original draft. All authors reviewed and edited the final manuscript.

## Funding

This research was funded by research core funding P1-0170 from the Slovenian Research Agency and a donation agreement with Sanofi, Shire d.o.o. (now part of Takeda), and Takeda Pharmaceuticals d.o.o. The APC was funded by Slovenian Research Agency (research core funding P1-0170). The funders/donators had no role in the design of the study; in the collection, analysis, or interpretation of data; in the writing of the manuscript; or in the decision to publish the results.

## Conflict of interest

TL received travel and accommodation funding from Takeda. BV received speakers’ fees and consultancy honoraria from Sanofi-Genzyme, Takeda, Amicus, Chiesi, and Greenovation Biotech GmbH, and is a member of the EU Advisory Board of Fabry Registry sponsored by Sanofi Genzyme. ACV received speakers’ fees, travel, and accommodation funding from Sanofi-Genzyme and Takeda. KTP received travel and accommodation funding from Sanofi Genzyme and speakers’ fees from Takeda.

## Publisher’s note

All claims expressed in this article are solely those of the authors and do not necessarily represent those of their affiliated organizations, or those of the publisher, the editors and the reviewers. Any product that may be evaluated in this article, or claim that may be made by its manufacturer, is not guaranteed or endorsed by the publisher.
