# Supplementary material for: Urinary-derived extracellular vesicles reveal a distinct microRNA signature associated with the development and progression of Fabry nephropathy
Source: Front Med (Lausanne). 2023 Mar 23;10:1143905. doi: 10.3389/fmed.2023.1143905 (PMC10076752; doi:10.3389/fmed.2023.1143905)
Supplement: Supplementary file 1 [file Table_1.docx]

Supplementary Material

Urinary-derived extracellular vesicles reveal a distinct microRNA signature associated with the development and progression of Fabry nephropathy

**Tina Levstek, Bojan Vujkovac, Andreja Cokan Vujkovac, Katarina Trebušak Podkrajšek***

*** Correspondence:** Prof. Katarina Trebušak Podkrajšek, PhD, ErCLG, EuSpLM

E-Mail: katarina.trebusakpodkrajsek@mf.uni-lj.si

**Supplementary Table S1.** Variants in the *GLA* gene (reference sequence NM_000169.2) and clinical characteristics of the included Fabry patients.

| Genetic variant | | Gender | Rate of progression | HCMP | Stroke | FD symptoms | α-gal A activity | LysoGb3 (ng/mL) | DST |  |
| --- | --- | --- | --- | --- | --- | --- | --- | --- | --- | --- |
| Discovery cohort | | | | | | | | | |  |
| c.125T>G | p.Met42Arg | M | progressive nephropathy | yes | yes | yes | 0.7 | 13.9 | yes | |
| p.Ile270Met | c.810T>G | M | stable renal function | yes | no | yes | 1.3 | 36.1 | yes | |
| p.358delGlu | c.1072_1074delGAG | M | progressive nephropathy | yes | yes | yes | 0.5 | 32.2 | yes | |
| p.Arg49Pro | c.146G>C | M | stable renal function | no | no | yes | 0.7 | 12.0 | yes | |
| p.Asn272Ser | c.815A>G | M | stable renal function | yes | no | yes | 0.6 | 14.2 | yes | |
| p.Arg363Pro | c.1088G>C | M | stable renal function | yes | no | yes | 1.2 | NA | yes | |
| p.Arg342Gln | c.1025G>A | M | progressive nephropathy | yes | no | yes | 7.0 | 14.5 | yes | |
| p.Asn272Ser | c.815A>G | M | stable renal function | no | no | yes | 0.7 | 30.9 | yes | |
| p.Asn272Ser | c.815A>G | M | progressive nephropathy | yes | no | yes | 0.4 | NA | yes | |
| p.Arg227Ter | c.679C>T | M | progressive nephropathy | yes | no | yes | 0.2 | 14.0 | yes | |
| Validation cohort | | | | | | | | | |  |
| p.Arg363Pro | c.1088G>C | F | progressive nephropathy | yes | no | yes | 4.6 | NA | yes | |
| p.Arg363Pro | c.1088G>C | F | progressive nephropathy | yes | no | yes | 22.9 | NA | yes | |
| p.Asn272Ser | c.815A>G | F | stable renal function | yes | no | yes | 16.2 | 5.5 | yes | |
| p.Arg227Ter | c.679C>T | M | progressive nephropathy | yes | no | yes | 0.2 | NA | yes | |
| p.Asn272Ser | c.815A>G | M | stable renal function | no | no | yes | 0.7 | 33.5 | yes | |
| p.Asn272Ser | c.815A>G | F | stable renal function | yes | no | yes | 22.1 | 5.6 | yes | |
| p.Ile270Met | c.810T>G | F | stable renal function | yes | yes | yes | 35.4 | 5.5 | yes | |
| p.Asn272Ser | c.815A>G | M | stable renal function | yes | no | yes | 0.6 | 11.0 | yes | |
| p.Leu180Phe | c.540G>C | F | progressive nephropathy | yes | yes | yes | 69.0 | <53 | yes | |
| p.358delGlu | c.1072_1074delGAG | M | progressive nephropathy | yes | yes | yes | 0.5 | 36.3 | yes | |
| p.Arg227Ter | c.679C>T | M | progressive nephropathy | no | no | yes | 1.2 | 6.2 | yes | |
| p.Asn272Ser | c.815A>G | F | stable renal function | yes | no | yes | 30.9 | 6.8 | no | |
| p.Ile270Met | c.810T>G | M | stable renal function | yes | no | yes | 1.3 | 40.0 | yes | |
| p.Asn272Ser | c.815A>G | F | stable renal function | no | no | yes | 33.3 | 2.7 | no | |
| p.Asn272Ser | c.815A>G | F | stable renal function | no | no | yes | 19.2 | 5.5 | no | |
| p.Asn272Ser | c.815A>G | F | progressive nephropathy | no | no | yes | 39.8 | 8.0 | no | |
| p.Arg342Gln | c.1025G>A | M | progressive nephropathy | yes | no | yes | 7.0 | NA | yes | |
| p.Glu87Asp | c.261_278del18 | F | stable renal function | no | no | yes | 25.0 | 8.2 | no | |
| p.Asn272Ser | c.815A>G | F | stable renal function | no | no | yes | 21.7 | 7.8 | no | |
| p.Asn272Ser | c.815A>G | F | stable renal function | yes | no | yes | 25.8 | 5.8 | yes | |
| p.Arg363Pro | c.1088G>C | M | stable renal function | yes | no | yes | 1.2 | 5.8 | yes | |
| p.Glu87Asp | c.261_278del18 | F | stable renal function | no | no | yes | 69.7 | 2.8 | no | |
| p.Arg49Pro | c.146G>C | F | stable renal function | yes | no | yes | 73.6 | 3.9 | no | |
| p.Arg49Pro | c.146G>C | M | stable renal function | no | no | yes | 0.7 | 17.0 | yes | |
| p.Arg342Gln | c.1025G>A | F | stable renal function | yes | no | yes | 60.1 | 6.1 | no | |
| p.Arg363Pro | c.1088G>C | F | stable renal function | yes | no | yes | 38.1 | 4.6 | no | |
| p.Arg363Pro | c.1088G>C | F | stable renal function | no | no | no | 13.7 | 5.3 | no | |
| p.Asn272Ser | c.815A>G | F | stable renal function | no | no | yes | 28.6 | 5.2 | no | |
| p.Arg227Ter | c.679C>T | F | stable renal function | no | no | yes | 53.0 | 5.5 | no | |
| p.Cys63Tyr | c.188G>C | F | stable renal function | no | no | yes | 37.6 | 7.6 | no | |
| p.Asn272Ser | c.815A>G | F | stable renal function | no | no | yes | 34.4 | 3.3 | no | |
| p.Asn272Ser | c.815A>G | F | stable renal function | no | no | yes | 15.9 | 6.6 | no | |
| p.Arg227Ter | c.679C>T | F | stable renal function | no | no | no | 45.5 | NA | no | |

Hypertrophic cardiomyopathy (HCMP) was assessed by echocardiography and/or cardiac MRI. Stroke was confirmed by appropriate imaging assessment. Characteristic Fabry disease (FD) symptoms were defined when present Fabry neuropathic pain, angiokeratoma, and/or cornea verticillata. α-galactosidase A (α-gal A) was measured in leukocytes and is reported as % of the mean of the reference range. Globotriaosylsphingosine (LysoGb3) was measured in serum. DST, disease specific therapy; NA, not available.

**Supplementary Table S2.** Summary of linear mixed models with time and group as fixed effects, subject as a random intercept, and log-transformed miRNA expression as a dependent variable.

| miR-30a-5p | | | | | | | | |
| --- | --- | --- | --- | --- | --- | --- | --- | --- |
| Fixed effects | | | | | | Random effects | | |
|  | Estimate | St. error | df | t value | *p-*value |  | Variance | St. dev. |
| Intercept | 0.802 | 0.085 | 37.9 | 9.456 | **<0.001** | Subject  (intercept) | 0.040 | 0.199 |
| Time | 0.004 | 0.005 | 118.4 | 0.742 | 0.459 |  |  |  |
| Group | 0.349 | 0.094 | 32.1 | 3.729 | **<0.001** | Residual | 0.055 | 0.234 |
| miR-222-3p | | | | | | | | |
| Fixed effects | | | | | | Random effects | | |
|  | Estimate | St. error | df | t value | *p-*value |  | Variance | St. dev. |
| Intercept | 0.041 | 0.110 | 34.2 | 0.369 | 0.715 | Subject  (intercept) | 0.082 | 0.287 |
| Time | −0.008 | 0.005 | 118.8 | −1.541 | 0.126 |  |  |  |
| Group | −0.049 | 0.124 | 31.6 | −0.395 | 0.695 | Residual | 0.048 | 0.220 |
| miR-204-5p | | | | | | | | |
| Fixed effects | | | | | | Random effects | | |
|  | Estimate | St. error | df | t value | *p-*value |  | Variance | St. dev. |
| Intercept | 0.880 | 0.097 | 33.6 | 9.064 | **<0.001** | Subject  (intercept) | 0.066 | 0.256 |
| Time | −0.012 | 0.004 | 118.6 | −2.836 | **0.005** |  |  |  |
| Group | 0.273 | 0.110 | 31.5 | 2.483 | **0.019** | Residual | 0.031 | 0.176 |
| miR-22-5p | | | | | | | | |
| Fixed effects | | | | | | Random effects | | |
|  | Estimate | St. error | df | t value | *p-*value |  | Variance | St. dev. |
| Intercept | −0.337 | 0.073 | 37.3 | −4.599 | **<0.001** | Subject  (intercept) | 0.028 | 0.166 |
| Time | 0.007 | 0.005 | 121.1 | 1.390 | 0.167 |  |  |  |
| Group | −0.125 | 0.080 | 30.9 | −1.559 | 0.129 | Residual | 0.048 | 0.220 |
| miR-21-5p | | | | | | | | |
| Fixed effects | | | | | | Random effects | | |
|  | Estimate | St. error | df | t value | *p-*value |  | Variance | St. dev. |
| Intercept | 1.393 | 0.112 | 34.3 | 12.402 | **<0.001** | Subject  (intercept) | 0.080 | 0.283 |
| Time | 0.002 | 0.006 | 118.8 | 0.397 | 0.692 |  |  |  |
| Group | −0.216 | 0.126 | 30.9 | −1.721 | 0.095 | Residual | 0.066 | 0.258 |
| miR-10b-5p | | | | | | | | |
| Fixed effects | | | | | | Random effects | | |
|  | Estimate | St. error | df | t value | *p-*value |  | Variance | St. dev. |
| Intercept | 0.546 | 0.130 | 33.4 | 4.203 | **<0.001** | Subject  (intercept) | 0.120 | 0.346 |
| Time | −0.007 | 0.005 | 118.5 | −1.308 | 0.193 |  |  |  |
| Group | 0.485 | 0.147 | 31.6 | 3.298 | **0.002** | Residual | 0.048 | 0.219 |
| let-7i-5p | | | | | | | | |
| Fixed effects | | | | | | Random effects | | |
|  | Estimate | St. error | df | t value | *p-*value |  | Variance | St. dev. |
| Intercept | −0.380 | 0.058 | 47.3 | −6.561 | **<0.001** | Subject  (intercept) | 0.009 | 0.096 |
| Time | 0.004 | 0.005 | 125.1 | 0.731 | 0.466 |  |  |  |
| Group | −0.031 | 0.061 | 32.9 | −0.511 | 0.613 | Residual | 0.056 | 0.237 |

**Bold**indicates *p* < 0.05 for fixed effects. df, degrees of freedom.

**Supplementary Table S3.** Top 20 genes identified by 11 topological analysis methods for genes targeted by upregulated miRNAs.

| Betweenness | BottleNeck | Closeness | Degree | DMNC | EcCentricity | EPC | MCC | MNC | Radiality | Stress |
| --- | --- | --- | --- | --- | --- | --- | --- | --- | --- | --- |
| *MYC* | *EGFR* | *MYC* | *MYC* | *ALDH1A1* | *CKAP5* | *EGFR* | *MYC* | *MYC* | *MYC* | *MYC* |
| *EGFR* | *MYC* | *EGFR* | *EGFR* | *MUC1* | *FOS* | *STAT3* | *STAT3* | *EGFR* | *EGFR* | *EGFR* |
| *ESR1* | *SOX2* | *PTEN* | *PTEN* | *CCL20* | *CCT5* | *ESR1* | *HIF1A* | *PTEN* | *PTEN* | *PTEN* |
| *PTEN* | *SKP1* | *ESR1* | *ESR1* | *TGFBI* | *VHL* | *PTEN* | *VEGFA* | *ESR1* | *ESR1* | *ESR1* |
| *HSPA8* | *ESR1* | *STAT3* | *STAT3* | *SELE* | *MUC1* | *MYC* | *PTEN* | *STAT3* | *STAT3* | *STAT3* |
| *CUL3* | *PTEN* | *HIF1A* | *VEGFA* | *SOCS5* | *E2F3* | *VEGFA* | *EGFR* | *VEGFA* | *HIF1A* | *BRCA1* |
| *BRCA1* | *BRCA1* | *BRCA1* | *HIF1A* | *BMF* | *ANKRD28* | *BRCA1* | *ESR1* | *HIF1A* | *MDM2* | *HSPA8* |
| *STAT3* | *CUL3* | *VEGFA* | *BRCA1* | *FASLG* | *SEC24C* | *HIF1A* | *FKHRL1* | *BRCA1* | *BRCA1* | *MDM2* |
| *MDM2* | *UBE2D3* | *MDM2* | *SOX2* | *TNFRSF11B* | *REST* | *SOX2* | *FOS* | *SOX2* | *VEGFA* | *HIF1A* |
| *VCP* | *AGO2* | *SOX2* | *MDM2* | *CD69* | *HDLBP* | *IL1B* | *IGF1R* | *MDM2* | *SOX2* | *CUL3* |
| *HIF1A* | *ERBB4* | *PIK3R1* | *FKHRL1* | *REV1* | *PTBP3* | *PIK3R1* | *CDKN1B* | *FOXO3* | *HSPA8* | *VEGFA* |
| *SOX2* | *HNRNPK* | *HSPA8* | *IL1B* | *JAG1* | *SOCS3* | *MMP9* | *SOX2* | *IL1B* | *PIK3R1* | *SOX2* |
| *RAB11A* | *VCP* | *FOXO3* | *PIK3R1* | *CCL1* | *APPL1* | *KIT* | *MDM2* | *PIK3R1* | *FOXO3* | *PIK3R1* |
| *YAP1* | *FKHRL1* | *KIT* | *CD4* | *PTX3* | *ARF4* | *CD4* | *CCNA2* | *CD4* | *CCNA2* | *HNRNPK* |
| *HNRNPK* | *RAB11A* | *CCNA2* | *MMP9* | *IL6R* | *SP1* | *FOS* | *MMP9* | *MMP9* | *KIT* | *YAP1* |
| *VEGFA* | *VEGFA* | *DICER1* | *KIT* | *HRK* | *IRS4* | *MDM2* | *BRCA1* | *KIT* | *DICER1* | *DICER1* |
| *SKP1* | *SMAD7* | *FOS* | *FOS* | *PKNOX1* | *CCR7* | *FOXO3* | *TGFB1* | *FOS* | *SP1* | *VCP* |
| *DDX3X* | *ARID1A* | *CD4* | *HSPA8* | *ADAM17* | *RNF6* | *SP1* | *IL1B* | *HSPA8* | *CDKN1B* | *FOXO3* |
| *PIK3R1* | *VAPB* | *SP1* | *CCNA2* | *CD47* | *SETD2* | *MMP2* | *MMP2* | *CCNA2* | *FOS* | *SKP1* |
| *DICER1* | *SEC63* | *CDKN1B* | *DICER1* | *AGGF1* | *AGO4* | *CDKN1B* | *CD4* | *DICER1* | *SKP1* | *DDX3X* |

DMNC, Density of Maximum Neighborhood Component; EPC, Edge Percolated Component; MCC, Maximal Clique Centrality; MNC, Maximum Neighborhood Component.

**Supplementary Table S4.** Top 20 genes identified by 11 topological analysis methods for genes targeted by downregulated miRNAs.

| Betweenness | BottleNeck | Closeness | Degree | DMNC | EcCentricity | EPC | MCC | MNC | Radiality | Stress |
| --- | --- | --- | --- | --- | --- | --- | --- | --- | --- | --- |
| *TP53* | *CTNNB1* | *TP53* | *TP53* | *PAK1IP1* | *ATP6V1V1* | *TP53* | *PTEN* | *TP53* | *TP53* | *TP53* |
| *CTNNB1* | *EGFR* | *CTNNB1* | *CTNNB1* | *REXO4* | *MRE11A* | *CTNNB1* | *TP53* | *CTNNB1* | *CTNNB1* | *CTNNB1* |
| *EGFR* | *TP53* | *EGFR* | *EGFR* | *SHOC2* | *SON* | *EGFR* | *JUN* | *EGFR* | *EGFR* | *EGFR* |
| *HSP90AA1* | *HSP90AA1* | *HSP90AA1* | *PTEN* | *PPP1R15B* | *NR3C2* | *PTEN* | *CASP3* | *PTEN* | *HSP90AA1* | *HSP90AA1* |
| *PTEN* | *YWHAE* | *PTEN* | *HSP90AA1* | *MAK16* | *SEPT7* | *HSP90AA1* | *BCL2L1* | *HSP90AA1* | *PTEN* | *PTEN* |
| *NOTCH1* | *SRSF1* | *JUN* | *JUN* | *UTP11L* | *CACNG2* | *JUN* | *CTNNB1* | *JUN* | *NOTCH1* | *JUN* |
| *JUN* | *MAPK1* | *NOTCH1* | *NOTCH1* | *BCL2L2* | *ERRFI1* | *NOTCH1* | *EGFR* | *NOTCH1* | *JUN* | *NOTCH1* |
| *HSPA5* | *JUN* | *SIRT1* | *CASP3* | *RRP15* | *LTN1* | *CASP3* | *HSP90AA1* | *CASP3* | *SIRT1* | *HSPA5* |
| *MAPK1* | *PTEN* | *CASP3* | *SIRT1* | *FRS2* | *EPHA2* | *SIRT1* | *MCL1* | *SIRT1* | *CASP3* | *MAPK1* |
| *SIRT1* | *HNRNPA2B1* | *MAPK1* | *MAPK1* | *RRP36* | *C17orf64* | *CDKN2A* | *CDKN2A* | *PIK3CA* | *CDKN2A* | *SIRT1* |
| *PPP1CC* | *LOX* | *CDKN2A* | *PIK3CA* | *CCNJ* | *CNOT1* | *MAPK1* | *NOTCH1* | *MAPK1* | *MAPK1* | *CASP3* |
| *CDKN2A* | *UBE2D3* | *CREB1* | *CDKN2A* | *CCNY* | *CDH2* | *PIK3CA* | *BCL2L11* | *CREB1* | *CREB1* | *CDKN2A* |
| *ACTG1* | *EZR* | *HSPA5* | *HSPA5* | *TNFRSF10B* | *PTEN* | *IL1B* | *IGF1R* | *CDKN2A* | *HSPA5* | *ACTG1* |
| *UBE2I* | *UBE2I* | *PIK3CA* | *CREB1* | *AP1S3* | *DPF2* | *CREB1* | *IL1B* | *HSPA5* | *ACTG1* | *CREB1* |
| *PIK3CA* | *KIF5B* | *ACRG1* | *IL1B* | *EPG5* | *BRWD1* | *IGF1R* | *CREB1* | *IL1B* | *PIK3CA* | *PIK3CA* |
| *CREB1* | *SREBF1* | *IL1B* | *CD44* | *WDR74* | *GJA1* | *ABL1* | *MAPK8* | *CD44* | *XPO1* | *UBE2I* |
| *EEF2* | *NR3C1* | *YWHAZ* | *UBE2I* | *PRIM1* | *BRWD3* | *CD44* | *PIK3CA* | *ACTG1* | *YWHAZ* | *AGO2* |
| *CASP3* | *GABARAPL2* | *XPO1* | *ACTG1* | *WDR75* | *MICAL1* | *MAPK8* | *SIRT1* | *ABL1* | *IL1B* | *PPP1CC* |
| *BPTF* | *PSMD7* | *IGF1R* | *ABL1* | *SLC25A36* | *CALB2* | *IRS1* | *MAPK1* | *UBE2I* | *IGF1R* | *EEF2* |
| *YWHAZ* | *EEF2* | *YWHAE* | *IGF1R* | *ANK1* | *BRD2* | *JAK2* | *JAK2* | *IGF1R* | *YWHAE* | *POLR2B* |

DMNC, Density of Maximum Neighborhood Component; EPC, Edge Percolated Component; MCC, Maximal Clique Centrality; MNC, Maximum Neighborhood Component.
